# Renal lymphangiectasia

**DOI:** 10.4322/acr.2021.399

**Published:** 2022-09-23

**Authors:** Sangamitra Rajasekaran, Mayur Parkhi, Ravi Kanojia, Aravind Sekar, Ritambhra Nada

**Affiliations:** 1 Post Graduate Institute and Medical Education and Research, Department of Pathology, Chandigarh, India; 2 Post Graduate Institute and Medical Education and Research, Department Histopathology, Chandigarh, India; 3 Post Graduate Institute and Medical Education and Research, Department of Pediatric Surgery, Chandigarh, India

**Keywords:** Kidney, Lymphangioma, Diagnosis, Differential, Immunohistochemistry

Renal lymphangiectasia (RL), also known as renal lymphangiomatosis, is a rare benign condition characterized by ectasia of peripelvic, perirenal and intrarenal lymphatic vessels. It accounts for approximately 1% of all lymphangiomas.^1^ The pathophysiology of renal lymphangiectasia remains unclear. However, it is hypothesized that the failure of draining into the larger retroperitoneal lymphatic channels causes abnormal cystic dilatation of the peripelvic, perirenal and intrarenal lymphatic ducts.^2^ RL can manifest at any age, with males and females equally affected. Lymphangiectasia can involve both kidneys, albeit unilateral involvement is common. Patients may be asymptomatic or present with flank pain, abdominal distention, lower limb oedema, hematuria and hypertension. Extreme presentation such as renal failure has also been documented. Due to the classical imaging features, computed tomography (CT) scan becomes the best diagnostic modality for the diagnosis, which can be confirmed by aspiration of chylous fluid.^3^ In the pediatric patient, the differential diagnosis includes cystic diseases of the kidney, nephroblastomatosis, and hydronephrosis with perinephric urinoma. Depending on the presentation, the management varies, including conservative, percutaneous aspiration, marsupialization, and nephrectomy.^4^

We describe gross and microscopic features of renal lymphangiectasia in a 4-year-old male child who presented with gradually progressive abdominal distension for one month with accompanying vague flank pain and fatigue. No hematuria or bladder bowel complaints were noted. Family and perinatal history were not significant. An ill-defined large mass of approximately 20x10cm occupying the left flank and hypochondrium was palpable on bimanual palpation. The renal function test was within normal limits for this age. Ultrasonography (USG) abdomen revealed bilateral multi-loculated, anechoic, cystic lesions in the perirenal and parapelvic region. Raised cortical echoes indicated the loss of corticomedullary distinction. CT scan revealed bilateral non-enhancing multiloculated cystic collection in the perirenal and parapelvic locations. Exploratory laparotomy showed a well-defined thick-walled sac of 20x15cm encasing the left kidney. Intraoperatively, the left kidney was hard to feel, and the architecture was distorted.

**Figure 1:**
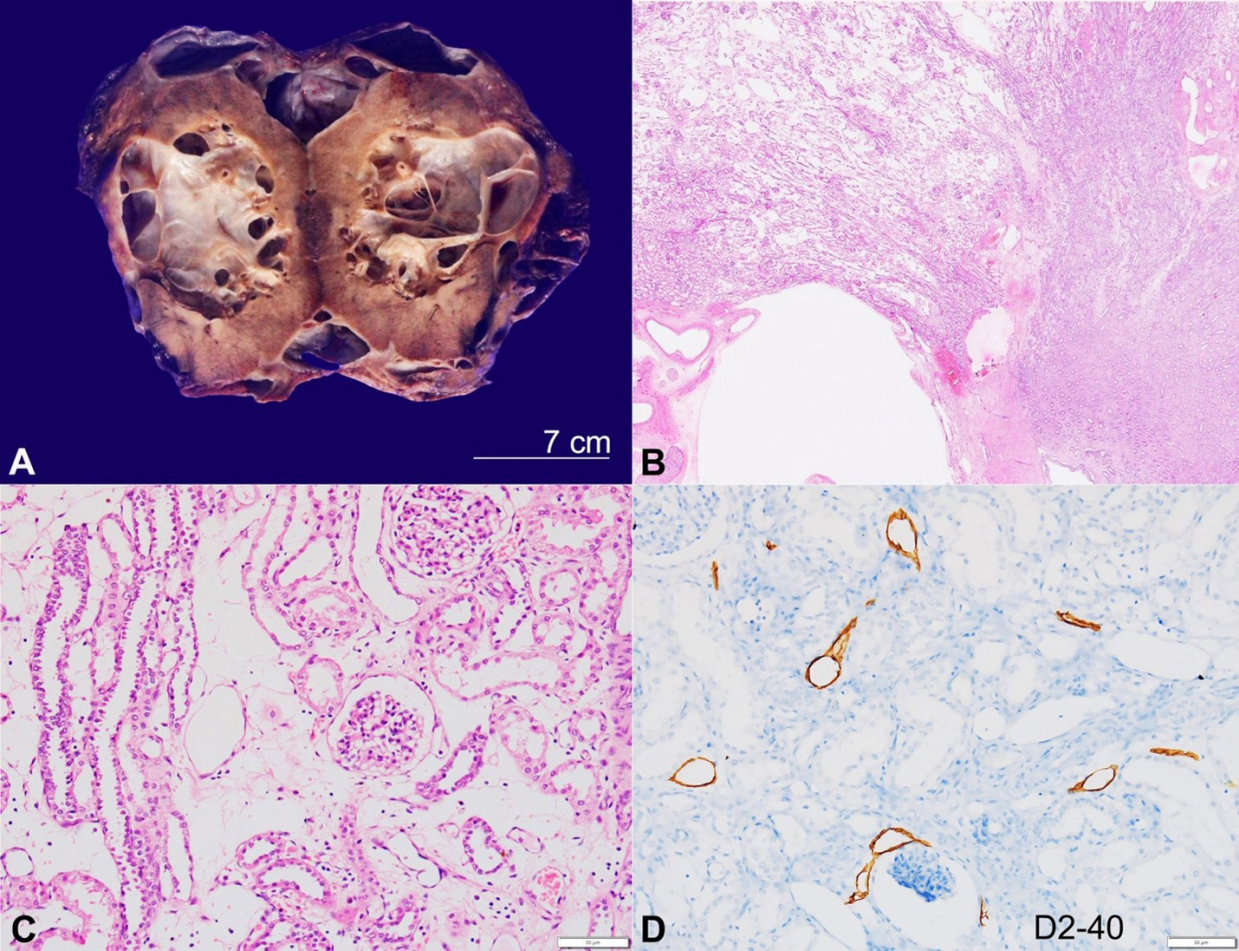
**A** - Gross view of the cut surface of a left kidney showing multiloculated cyst involving the renal cortex, medulla and the perinephric region. **B**, **C** and **D** Microphotographs of the kidney. **B** - Scanning microphotograph depicting various cysts, distorting normal renal parenchymal architecture (H&E, 10x); **C** - The interstitium showing edema along with few dilated lymphatic channels (H&E, 200x); **D** - D2-40 immunostaining highlights dilated lymphatic channels in the renal parenchyma (200x).

A Left nephroureterectomy was done and submitted for histopathological evaluation. The kidney with perinephric fat measures 15x9x5.5cm, and the ureter was 5cm in length. The perinephric fat showed multiple collapsed cysts that enclosed the whole kidney ranging in size from 4 to 8cm in the largest dimension. The cut surface of the kidney exhibited well-demarcated multiloculated cysts in the cortex and medulla. The cysts are of variable size measuring 0.5 to 4cm in maximum dimension and shows thickness of 0.2cm and intervening thin septa (Figure 1A). The luminal aspect appeared smooth, contained brownish serous fluid, and did not show any papillary excrescences or growth. The adjacent spared kidney showed indistinct cortico-medullary junction and focal thinning of the cortex (0.3 to 0.4cm). Microscopically, numerous and variably sized cystic spaces were present in the cortex, medulla, pelvis, and perinephric fat (Figure 1B). Glomeruli and tubules in the intervening renal parenchyma showed no abnormal pathology on microscopy; however, the interstitium appears oedematous containing dilated lymphatic channels (Figure 1C). Cystic spaces were lined by a discontinuous layer of flat endothelial cells as highlighted by D2-40 (Figure 1D), CD31, and CD34 immunostains. Neuromatoid hyperplasia was seen in the sections from renal pelvis. Following clinical-radio-pathological correlation, a diagnosis of renal lymphangiectasia was offered. The patient was on close imaging follow-up to keep track of the right kidney.
